# Radiotherapy of large target volumes in Hodgkin's lymphoma: normal tissue sparing capability of forward IMRT versus conventional techniques

**DOI:** 10.1186/1748-717X-5-33

**Published:** 2010-05-11

**Authors:** Laura Cella, Raffaele Liuzzi, Mario Magliulo, Manuel Conson, Luigi Camera, Marco Salvatore, Roberto Pacelli

**Affiliations:** 1 Institute of Biostructures and Bioimages, National Council of Research (CNR), Via Pansini 5, 80131, Naples, Italy; 2 Department of Diagnostic Imaging and Radiation Oncology, University "Federico II" of Naples, Via Pansini 5, 80131, Naples, Italy

## Abstract

**Background:**

This paper analyses normal tissue sparing capability of radiation treatment techniques in Hodgkin's lymphoma with large treatment volume.

**Methods:**

10 patients with supradiaphragmatic Hodgkin's lymphoma and planning target volume (PTV) larger than 900 cm^3 ^were evaluated. Two plans were simulated for each patient using 6 MV X-rays: a conventional multi-leaf (MLC) parallel-opposed (AP-PA) plan, and the same plan with additional MLC shaped segments (forward planned intensity modulated radiation therapy, FPIMRT). In order to compare plans, dose-volume histograms (DVHs) of PTV, lungs, heart, spinal cord, breast, and thyroid were analyzed. The Inhomogeneity Coefficient (IC), the PTV receiving 95% of the prescription dose (V95), the normal tissue complication probability (NTCP) and dose-volume parameters for the OARs were determined.

**Results:**

the PTV coverage was improved (mean V95_AP-PA _= 95.9 and IC_AP-PA _= 0.4 vs. V95_FPIMRT _= 96.8 and IC_FPIMRT _= 0.31, *p *≤ 0.05) by the FPIMRT technique compared to the conventional one. At the same time, NTCPs of lung, spinal cord and thyroid, and the volume of lung and thyroid receiving ≥ 30 Gy resulted significantly reduced when using the FPIMRT technique.

**Conclusions:**

The FPIMRT technique can represent a very useful and, at the same time, simple method for improving PTV conformity while saving critical organs when large fields are needed as in Hodgkin's lymphoma.

## Background

Radiation treatment and antiblastic chemotherapy of Hodgkin's lymphoma (HL) is a proven curative therapeutic strategy capable of curing the vast majority of patients. Radiotherapy in Hodgkin's lymphoma is very often characterized by fields encompassing different body sites. The great variability of thickness and density in the irradiated tissues makes it difficult to achieve a homogeneous distribution of the dose. Moreover, the low average age of HL patients, in the cases in which a large volume needs to be irradiated, makes these patients' population particularly at risk of developing late side effects and secondary neoplasms. The irradiation of the thyroid region, for instance, induces a 50% risk of developing hypothyroidism and a 20% risk to develop thyroid nodules [1-3]. Radiation dose and irradiated volume of the thyroid gland correlate with the incidence of hypothyroidism. In particular, the volume of gland receiving a dose greater than 30 Gy has been shown to significantly impact on the TSH peak [4]. Irradiated volume at given dose levels can be also related to late cardiac and pulmonary toxicity [5,6]. For example, the risk of grade 3 late lung toxicity has been found to be 38% or 4% depending on whether the volume receiving 25 Gy is larger or smaller than 30% respectively [7]. Several papers have reported an increased risk of breast cancer in girls and young women among HL patients: breast cancer represents 6.3 to 9% of all secondary cancers occurring after HL treatment [8]. Higher radiation doses might increase the risk of developing breast cancer. Tailoring radiotherapy to eliminate as much breast tissue as possible from the radiation field may reduce this risk [9]. All these issues have to be carefully considered by the radiation oncologists approaching the therapeutic strategy in HL while the medical physicists have to make every possible effort to optimize treatment plans.

Perhaps, due to the low doses used in the treatment of this disease, only in recent years some efforts have been made to improve dose distribution in HL treatment plans. Several delivery techniques, such as intensity modulated radiation therapy (IMRT) techniques with or without inverse planning optimization and even three- dimensional proton radiotherapy, have been proposed in the literature [10-16]. All these techniques aim at achieving better homogeneity in target dose distribution and dose reduction to critical structures. However, there have been some discussions on IMRT techniques for large planning target volumes (PTVs) and their actual implementation due either to field size restrictions using dynamic multileaf collimators [17,18] or to the greater volume of normal tissue receiving low-to-moderate radiation doses and its related late radiation effects [19].

In this work we define and quantify the dosimetric advantages of a forward planned intensity modulated technique (FPIMRT) via segmented fields [20] for selected Hodgkin's lymphoma patients for whom large field irradiation is required. To this purpose we have simulated ten consecutive HL patients undergoing post chemotherapy involved field radiotherapy with PTV larger than 900 cm^3^. For each patient two treatment plans, a conventional parallel-opposed field plan and a FPIMRT plan, were retrospectively generated. Dose homogeneity in the target and normal tissue sparing capability were the main focus of our analysis.

## Methods

Ten patients with Hodgkin's disease who had received post chemotherapy radiotherapy at the Department of Radiotherapy of the University "Federico II" of Naples were retrospectively considered for the study. These patients had stage II disease requiring a large volume of irradiation. They represent about 6% of the patients treated in the last 9 years at our department. Patients and disease characteristics are shown in table 1. Mean age was 25.6 years (95% CI, 18.7-32.5). In all patients a continuous CT-scan was performed in supine position using vacuum locked mattress with the arms up above the head. Scans were acquired using 5-mm slices of a multislice scanner with the craniocaudal limits, generally 4 cm behind the target region.

**Table 1:** Patient and disease characteristics

Patient	Age	Gender	Stage	Disease sites	PTV (cm^3^)	Subfields
1	19	M	II-B	Mediastinum*, bilat. LCV and axill. nodes	2285.2	4
2	19	M	IV-AS	Mediastinum, bilat. LCV, SCV, and axill. nodes	2449.6	5
3	19	M	III-AS	Mediastinum, R axill. nodes	1262.9	3
4	25	M	III-BS	Mediastinum, bilat. LCV, SCV nodes, L axill. nodes	2168.7	4
5	41	F	IV-B	Mediastinum, bilat. SCV nodes	920.5	2
6	21	F	II-B	Mediastinum, bilat LCV nodes, L SCV nodes	2511.4	5
7	42	F	II-A	Antero-superior mediastinum †, bilat. axill. nodes, L SCV nodes	1657.1	4
8	34	F	II-A	Antero-superior mediastinum, L LCV nodes	970.1	3
9	18	F	II-A	Antero-superior mediastinum, R LCV nodes, bilat SCV nodes, R axill. nodes	1259.1	3
10	18	F	II-A	Antero-superior mediastinum, L LCV nodes, bilat. SCV nodes, L axill. nodes	1033.4	3

*Abbreviations*: PTV = planning target volume, M = male, F = female, R = right, L = left, LCV = laterocervical, SCV = sovraclavear, bilat. = bilateral, axill. = axillary.

* superior mediastinal nodes, aortic nodes, inferior mediastinal nodes, hilar nodes

† superior mediastinal nodes, aortic nodes.

CT images were electronically transferred to the Focal Ease 4.2 CT Simulation software (Computerized Medical System, Inc., St Louis, MO) for the contouring of target and critical organs (lung, spinal cord, heart, thyroid and, in women, breast). Target volumes and organs at risk were delineated by the same radiation oncologist (R.P.) and checked by a senior radiologist (L.Ca.).

Clinical target volume (CTV) included the nodal sites involved at the time of diagnosis. The nodal sites were delineated according to the modalities in use for three dimensional conformal radiotherapy (3D-CRT) in solid tumors. Namely, for the neck we referred to the internationally accepted guidelines of Gregoire *et al*. [21], to Mountain and Dresler [22] for the mediastinum, and to Dijkema *et al*. [23] for supraclavear and axillary nodes. Planning target volume (PTV) included CTV plus a 10 mm margin. For this study, we considered patients with a target volume larger than 900 cm^3 ^(all PTV volumes are reported in table 1).

Treatment planning was done by a 3-D planning system (XiO 4.4, Computerized Medical System, Inc., St Louis, MO). Two new treatment plans were on purpose generated for each patient: conventional anterior-posterior and posterior-anterior (AP-PA) plan and FPIMRT plan. Both plans were simulated using 6 MV X-rays with a dose rate of 200 MU/min, from Siemens Primus (Siemens Medical Systems, Erlanger, De) linear accelerator equipped with 29 pairs of double-focused multileaf collimator (MLC). A total dose of 30 Gy in 20 daily fractions of 1.5 Gy was planned. The same physicist performed all treatment plans. For both techniques, treatment plans were optimized to ensure, when possible, that 95% of the prescription dose was delivered at least to 95% of the PTV and, at the same time, with a maximum dose less than 120%.

The dose distribution was calculated using the Xio Multigrid Superposition algorithm [24] appropriate in the presence of heterogeneous tissues.

### Plan 1. Conventional Plan

In the AP and PA fields the MLC was shaped to the projection of the PTV in the beam's-eye view. The collimator was set to 0° or 270°, depending on the best MLC orientation for the optimal shielding. The prescription dose was specified at the centre of PTV. Field weightings were adjusted to achieve the maximum possible uniform distribution in the target volume. It must be stressed that conventional plans were not actually used for treating patients, but were generated to evaluate the overall advantages of the FPIMRT technique and to allow comparison with other techniques proposed in the literature [13,14].

### Plan 2. FPIMRT plan

**Figure 1:**
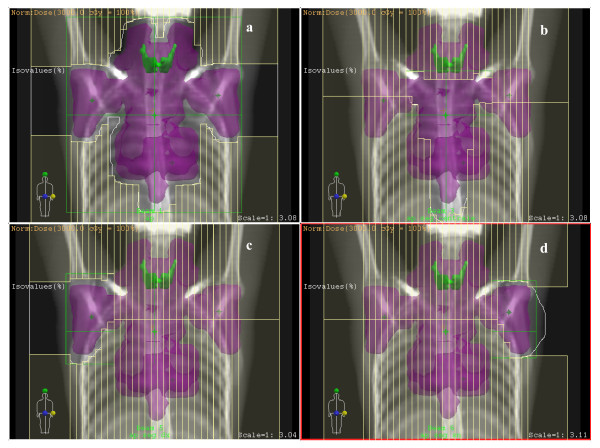
**FPIMRT portals**. Example of FPIMRT portals: a) main anterior-posterior field (AP); b) central AP subfield; c) right AP axillary subfield; d) left AP axillary subfield. The PTV is shown in magenta color and the thyroid gland in green.

In the FPIMRT technique a step-by-step iterative process inherent to forward planning was used as described elsewhere [10,20]. Briefly, the starting point was the conventional AP-PA plan. Then, additional MLC shaped subfields (segments) with the same AP-PA isocenter and gantry position were manually added. Two or more segments were used, with a maximum of 5, depending on the disease sites and target volume (see table 1 for details). In any case, we have always used segments with more than 7 monitor units (MUs) [25]. The prescription dose was specified at the centre of PTV for the AP and PA fields; for the MLC subfields the dose was prescribed at geometrical subfield center at isocenter depth. Figure 1 shows an example of one of the FPIMRT portals which consists of one main AP field (figure 1a) and three subfields (figures 1b, 1c, and 1d). In this example, 13 MUs were given for the mediastinal subfield and 10 MUs for each of the axillary subfields.

In order to achieve a better homogeneity in dose distribution and normal tissue sparing, the MLC positions and beam weightings were optimized by forward planning based on the 3D dose distribution as well as on dose-volume histograms (DVHs). DVHs were also used to evaluate the quality of the plan through dose volume constraints and target dose homogeneity. If performed by experienced physicist, the FPIMRT takes on average 20 minutes more than conventional planning process.

### Plan evaluation

In order to evaluate and compare plans, dose-volume histograms (DVHs) were computed for the target and critical organs. DVHs were assessed quantitatively, for each of the above plans and for all patients, by recording the minimum, maximum and mean doses. The percent of PTV volume within 95% (V95) isodose was also recorded.

The Inhomogeneity Coefficient (IC) [26] was calculated for each plan and for all patients using the following formula:

The meaning of IC is that a lesser value of IC indicates better dose homogeneity in the PTV. Furthermore, we recorded dose-volume parameters as the volume of lungs receiving at least 20 Gy (VL20) and 30 Gy (VL30) and the volume of the thyroid gland receiving at least 30 Gy (VT30).

DVHs were also used to predict normal tissue complication probabilities (NTCPs) for lungs, heart, spinal cord and thyroid. We used a NTCP tool in XiO based on Lyman's dose-response model [27] and the "effective volume method" introduced by Kutcher *et al*. [28]. The parameters for NTCP calculations (volume effect, slope, and tolerance doses) were taken from Burman *et al*. [29] and are shown in table 2. Because of the low doses involved in the planning procedure, we calculated NTCP corresponding to tolerance doses leading to 5% complication rates at 5 years (TD_5/5_), except for the lung for which we considered the tolerance dose leading to 50% complication rates (TD_50/5_).

**Table 2:** Parameters used in XIO NTCP tool

Organ	Size factor (*n*)	Slope (*m*)	TD_5/5_ (Gy)	TD_50/5_ (Gy)	End Point
Lung	0.87	0.18		24.5	Pneumonities
Heart	0.35	0.10	40		Pericardities
Spinal cord	0.05	0.175	47		Myelities/necrosis
Thyroid	0.22	0.26	45		Thyroidities

*Abbreviations*: TD_5/5 _= tolerance dose leading to 5% complication rates at 5 years, TD_50/5 _= tolerance dose leading to 50% complication rates at 5 years.

As a final point, in order to evaluate treatment efficiency, we compared the total MUs needed for the two different techniques.

### Statistical Analysis

After verifying that data were normally distributed (Shapiro-Wilk normality test), the two different planning techniques were compared by paired Student t test in order to verify the significance of differences in the mean outcomes of the treatment plans. Only for breast data (6 female patients) we used the median and the range to describe the dosimetric parameters and nonparametric techniques employed for analyzing them (Wilcoxon matched-pairs tests). A *p *value of 0.05 was taken for significance. Statistical analysis was performed with GraphPad Prism 5.00 (GraphPad Software, San Diego CA).

## Results

### Planning Target Volume Coverage

**Figure 2:**
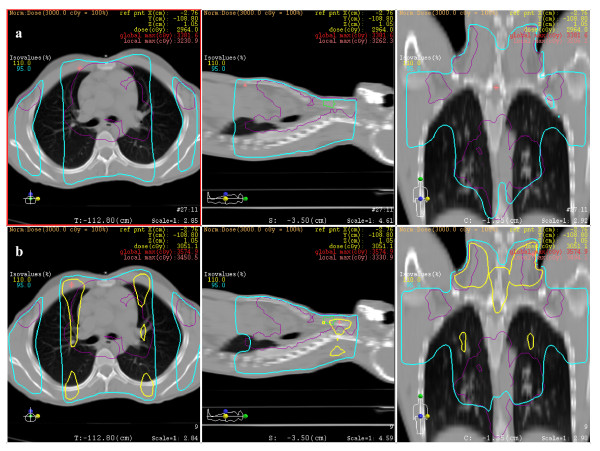
**Dose distributions**. Comparison of dose distribution of FPIMRT (a) vs. conventional (b) plans showing 110% (yellow line) and 95% (cyan line) isodoses in axial, sagittal and coronal sections.

Mean volume of the PTV was 1652 cm^3 ^(95% CI, 1191-2112). Mean dosimetric parameters for PTV were shown in table 3. Except for the minimal doses which were similar for the two techniques, all dosimetric parameters were significantly in favor of the FPIMRT plan. For all patients, PTV coverage and homogeneity have been improved when using FPIMRT technique compared with AP-PA technique. Figure 2 shows the comparative dose distribution in one of the patients.

**Table 3:** Mean dosimetric parameters and 95% confidence interval for PTV

Parameter	AP-PA	FPIMRT	*p*-value	IMRT plan **Goodman *et al*.**[13]
D_min _(%)	77 (75-79)	79 (77-82)	n.s.	76
D_max _(%)	118 (115-120)	111 (109-113)	< 10^-4^	120
D_mean _(%)	105 (104-105)	101 (100-102)	< 10^-4^	107
V95 (%)	95.9 (95.1-96.8)	96.8 (96.1-97.5)	0.05	98
IC	0.40 (0.37-0.42)	0.31 (0.29-0.34)	0.0002	-

*Abbreviations*: AP-PA = parallel opposed technique; FPIMRT = forward planned intensity modulated radiation therapy technique; D_min _= minimal dose, D_max_, = maximal dose; D_mean _= mean dose; IC = inhomogeneity coefficient, V95 = percent of PTV volume within 95% isodose, n.s. = not significant.

### Dose to Critical Organs

#### Lung

**Figure 3:**
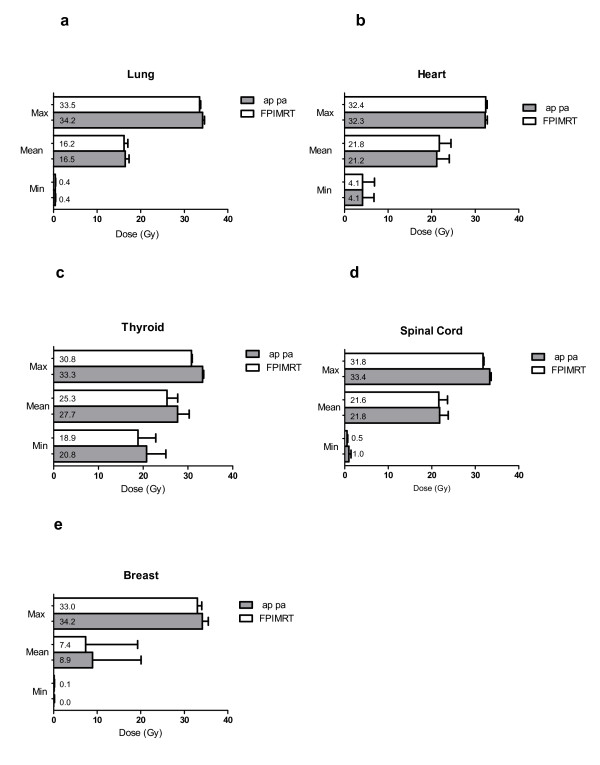
**Minimum, mean, and maximum doses**. Mean values of minimum, mean, and maximum doses for the AP-PA and the FPIMRT plans in a) lung; b) heart; c) thyroid; d) spinal cord; e) breast.

**Figure 4:**
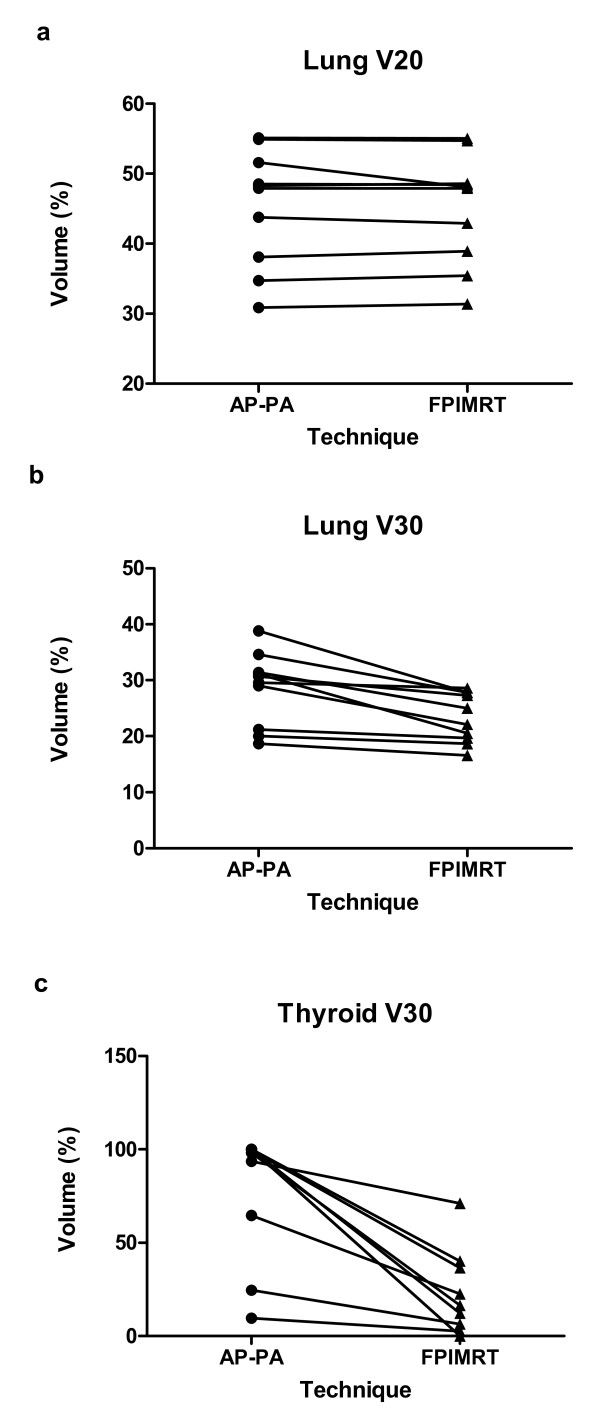
**Dose-volume parameters for lung and thyroid**. Lung V20 (a), lung V30 (b) and thyroid V30 (c) for the AP-PA and the FPIMRT plans.

The lung mean volume was 3127 cm^3 ^(95% CI, 2330-3924). As to the dose to the lung (figure 3a)), the mean values of minimum, maximum and mean doses were similar to both AP-PA and FPIMRT plans. As shown in table 4 and figure 4a and 4b, whereas the volume receiving a low dose (VL20) was unchanged, it is worth noting that in all FPIMRT plans the volume of lungs receiving at least 30 Gy (VL30) was significantly reduced (*p *= 0.002). Mean values of predicted NTCPs for lung corresponding to the tolerance dose TD_50/5 _are presented in table 5 for the two plans. The FPIMRT plan appears to have significantly reduced the NTCP (*p *= 0.03), and, consequently, the risk of late pneumonitis, compared to the conventional plan.

**Table 4:** Mean values and 95% confidence interval for OAR dose-volume parameters

Parameter	AP-PA	FPIMRT	*p*-value
VL20 (%)	45.4 (39.4-51.3)	45.1 (39.5-50.7)	n.s.
VL30 (%)	28.5 (23.8-33.2)	23.4 (20.2-26.6)	0.002
VT30 (%)	79.0 (54.2-103.7)	20.8 (4.5-37.1)	0.0005
VB20* (%)	21.2 (5.8-58.2)	20.7 (5.8-57.7)	n.s.

*Abbreviations*: VL20 = volume of lung receiving at least 20 Gy, VL30 = volume of lung receiving at least 30 Gy, VT30 = volume of thyroid receiving at least 30 Gy, VB20 = volume of breast receiving at least 20 Gy, other abbreviations as in table 3.

* Median and range

**Table 5:** Mean values and 95% confidence interval of predicted NTCPs (%)

		*NTCP(%)*	
**Organ**	**AP-PA**	***FPIMRT***	***p*-value**

Lung	7.6 (3.6-11.6)	6.2 (2.8-9.6)	0.03
Heart	0.3 (0-0.7)	0.3 (0-0.8)	n.s.
Spinal cord	2.1 (1.6-2.6)	1.6 (1.3-1.9)	0.02
Thyroid	11.6 (8.1-15.2)	7.6 (5.4-9.8)	0.0002

*Abbreviations*: NTCP normal tissue complication probability; other abbreviations as in table 3.

#### Heart

Mean volume of the heart was 570.9 cm^3 ^(95% CI, 460-681.8). Figure 3b shows the mean values of minimum, maximum and mean doses to the heart for both plans. Mean values of predicted NTCPs are reported in table 5. Comparing plan 1 and plan 2, the irradiation of the heart was comparable in the two techniques (same low NTCP and doses, *p *= 0.85).

#### Thyroid

Mean volume of the thyroid gland was 41.1 cm^3 ^(95% CI, 34.5-47.7). As to the dose to the thyroid, the average values of minimum, maximum and mean doses were significantly lower in the FPIMRT plan with a *p *value lower than 0.002 (figure 3c). All FPIMRT plans significantly succeeded in decreasing the VT30 parameter (figure 4c and table 4) compared with the conventional treatment (*p *= 0.0005). Furthermore, also the mean value of NTCP (table 5) for thyroid was significantly in favor of the FPIMRT treatment (*p *= 0.0002). From the results of these dosimetric parameters, thyroid toxicity was appreciably reduced when using the FPIMRT plan compared with the AP-PA plan.

#### Spinal Cord

As shown in figure 3d, the mean value of the maximum dose to the spinal cord is significantly reduced with the FPIMRT plan (*p *= 0.0003). Moreover, this plan succeeded in reducing the mean value of predicted NTCP reported in table 5 (*p *= 0.02).

#### Breast

Median volume of the breast was 1204 cm^3 ^(range 590.2 to 2392 cm^3^). As shown in figure 3e and in table 4, both AP-PA and FPIMRT plans delivered comparable radiation to the breast. No data were available for NTCP calculations.

### Monitor Units

The mean value of total Monitor Units was 165.1 (95% CI 161.6-168.6) and 190.8 (95% CI 181.8-199.8) with the conventional and FPIMRT treatments, respectively. Comparing plan 1 with plan 2, the mean per cent increase in MUs was 15.6%, that is, considering a dose rate of 200 MU/min, just less than 10 seconds of machine treatment increment.

## Discussion

Radiation treatment of Hodgkin's lymphoma is an efficient therapeutic modality that, coupled with antiblastic chemotherapy, can cure the large majority of patients [30,31]. Despite substantial advances in radiation treatment techniques in many areas, radiotherapy for HL is still delivered in a conventional way in most radiotherapy departments, and dose gradients that do not perfectly comply with general RT guidelines recommendations are often accepted.

Alternative delivery techniques with different complexity levels aimed at achieving better target coverage and critical structures sparing have been recently proposed.

A sliding window mantle technique [12], using dynamic MLC (dMLC) and electronic tissue compensation, succeeded in obtaining a better and more homogeneous target coverage in comparison with the conventional plan. However, the monitor unit number was increased by a factor of 3 in dMLC plan. Some investigators have proposed the use of IMRT for HL fields. Goodman *et al*. [13] used IMRT to irradiate lymphoma patients selected on the basis of either a large mediastinal treatment volume or because particularly at risk (reirradiation or previous antracyclin based treatment). The latter showed an improved target coverage and an amelioration in the pulmonary toxicity profile. Girinsky *et al*. [14] showed that, for mediastinal HL masses, IMRT achieves a better dose conformation and PTV coverage compared to 3D-CRT. Moreover, the heart, coronary arteries, esophagus, and spinal cord were more protected with IMRT plan, the only drawback being a greater volume of tissue receiving low doses compared to the conventional plan. Indeed the median dose delivered to the body increased seven folds. As the authors pointed out, this can be of concern in relation to the young age and long life expectation of HL patient population. Furthermore, IMRT technique becomes particularly complex in those cases in which large volumes have to be covered. Large IMRT fields cannot usually be implemented using available linacs because of issues related to MLC design [17]. In addition, because of the considerable cost and requirement of human resources, there are still many centers that have no adequate funds to implement this technique.

A simple forward planned IMRT technique has been suggested, in which dose conformation is obtained by combining MLC AP - PA fields and segments, with simple beam weighting modulation [10,11]. The authors describe better dose homogeneity and only assume a reduction in complication rate compared to conventional methods. In our clinical practice the FPIMRT is currently the standard technique for HL radiation treatment, regardless of the target dimensions.

The present report expands on the potential of the FPIMRT technique and extends the complexity of the analysis in order to evaluate and quantify the possible advantage of this technique vs. the conventional one in the case of large treatment fields in Hodgkin's lymphoma. Starting from an accurate and reproducible delineation of the target volume and of the OARs, the comparison was made considering normal tissue sparing capabilities. Dose volume constrains and NTCPs were the main focus of the evaluation. Indeed, in the past few years, the FPIMRT has been utilized to improve dosimetry in radiation therapy planning, and its general advantage on PTV coverage and homogeneity has been well documented. However, to our best knowledge, the advantage on normal tissue sparing in HL is only hypothesized and not quantified.

In addition, in this work we propose a reproducible way of drawing the target in HL patients following the nodal delineation suggested by other authors [21-23] for 3D conformal radiotherapy in solid tumors. Indeed, since the great variability in target definition represents a critical issue in the evaluation of different techniques [32], some standardization is needed.

As regards dose homogeneity and target coverage, we obtained good results with the FPIMRT technique compared to the conventional AP-PA treatment, being the dosimetric parameters for PTV significantly better with the FPIMRT plan. Indeed, adding segments in the right positions with appropriate weights allows to avoid, at the same time, hot and cold spot regions characteristic of the AP-PA treatment. As shown in table 3, all mean dosimetric parameters for PTV are similar to those obtained by other authors with full IMRT on large PTV [13]. We could not make a direct comparison on our patients since in our centre we don't have the suitable technology to perform IMRT for large treatment fields.

Despite the simplicity of the FPIMRT technique and the large PTVs considered, the obtained results were encouraging when we also consider doses to critical organs and the related toxicity rates. We found that the FPIMRT technique allows a reduction of normal tissue complication probability in all critical structures other than the heart for which both the NTCPs and the dosimetric parameters resulted comparable.

The appropriate parameters to be used to describe the probability of pulmonary toxicity are a matter of debate, and different predictive parameters have been proposed in literature [6,33,34] including the mean lung dose and the V10-V30. Considering our results, we can see that while the mean lung dose and V20 were similar for the two different techniques, V30 was significantly reduced with the FPIMRT plan. This result could indicate a lower pulmonary toxicity since radiation pneumonities rates seem to be correlated with a reduction in higher dose volume rather than with a reduction in lower dose volume [33]. If we compare our results for lungs with those obtained with IMRT on large volumes [13], we obtain a somewhat higher mean lung dose whereas, with the IMRT, V20 was greater.

The FPIMRT technique has also the advantage, when compared to the conventional one, of decreasing the maximum dose to the spinal cord (fig. 3d). However, no change was found for breast irradiation.

The results of our analysis are particularly striking when considering the thyroid gland: all dosimetric parameters and NTCP improved. Indeed, for all patients, V30 (figure 4c) resulted greatly reduced with a consequent lowering of hypothyroidism risk [4].

An indirect comparison of FPIMRT with full IMRT optimization suggests that, for smaller target volumes, full IMRT allows a better sparing of the heart and the coronary arteries from the high dose region [14]. Nevertheless, when larger target volumes are considered, because the advantages of full IMRT in heart sparing decrease [13] and the associated workload increases, this more sophisticated technique doesn't seem worthwhile. Another aspect that must be considered, especially in young patients, is the risk of induction of secondary malignancies which may result from larger low dose tissue volumes with IMRT [16].

As a whole, when considering target coverage improvement, OAR sparing capabilities, the ease of execution and delivery time, the use of the FPIMRT technique shows not only a definite improved performance when compared to the conventional AP-PA technique, but also represents a valid alternative when more sophisticated techniques are not available.

## Competing interests

The authors declare that they have no competing interests.

## Authors' contributions

LCe and RP conceived and designed the study. LCe, LCa, MC, MS and RP performed treatment planning procedure. RL, MM, RP and LCe analyzed the data. All authors participated in drafting and revising the manuscript. All authors have given their final approval of the manuscript.
